# The study of *METTL14*, *ALKBH5*, and *YTHDF2* in peripheral blood mononuclear cells from systemic lupus erythematosus

**DOI:** 10.1002/mgg3.1298

**Published:** 2020-06-25

**Authors:** Qing Luo, Jiayue Rao, Lu Zhang, Biqi Fu, Yang Guo, Zikun Huang, Junming Li

**Affiliations:** ^1^ Department of Clinical Laboratory The First Affiliated Hospital of Nanchang University Nanchang Jiangxi China; ^2^ Department of rheumatology The First Affiliated Hospital of Nanchang University Nanchang Jiangxi China

**Keywords:** *ALKBH5*, *MTEEL14*, systemic lupus erythematosus, *YTHDF2*

## Abstract

**Background:**

This study was aimed to explore the mRNA expression of m6A “writers” (*METTL3*, *MTEEL14*, and *WTAP*), “erasers” (*FTO* and *ALKBH5*), and “readers” (*YTHDF2*) in peripheral blood mononuclear cells (PBMCs) from systemic lupus erythematosus (SLE) patients and investigate the relation between their expressions with clinical features.

**Methods:**

In all, 54 SLE patients and 42 healthy controls (HC) were included in the current study. Quantitative reverse transcription‐polymerase chain reaction (qRT‐PCR) was used to investigate the mRNA expression of m6A “writers,” “erasers,” and “readers” in PBMCs from SLE patients and HC.

**Results:**

Decreased mRNA expression of *MTEEL14*, *ALKBH5*, and *YTHDF2* was observed in SLE patients compared with those in HC (*p* < .001). The decreased mRNA expression of *METTL14* was associated with white blood cell count (WBC) and monocyte count (M), this decreased mRNA expression of *ALKBH5* was associated with C‐reactive protein (CRP), neutrophil percentage (N%), lymphocyte percentage (L%), neutrophil–lymphocyte ratio (NLR), complement 3 (C3), and fever, and the decreased mRNA expression of *YTHDF2* was associated with L%, NLR, C3, and fever. In addition, there was a positive correlation between mRNA expression of *METTL14*, *ALKBH5*, and *YTHDF2* in PBMCs from SLE patients. Importantly, logistic regression analysis revealed that decreased mRNA expression of *YTHDF2* was a risk factor for SLE.

**Conclusion:**

Taken all together, our findings suggested decreased *YTHDF2* that was associated with disease activity may play an important role in the pathogenesis of SLE, *METTL14* and *ALKBH5* may be concomitantly decreased.

## Graphical abstract

SLE patients had lower mRNA expression of METTL14, ALKBH5 and YTHDF2 and logistic regression analysis revealed that decreased mRNA expression of YTHDF2 was a risk factor for SLE

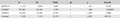

## 1 INTRODUCTION

Systemic lupus erythematosus (SLE) is the archetypal multisystem autoimmune disease characterized by unpredictable patterns of flares and remission. SLE patients are predominantly young women who suffer a marked loss of life expectancy and severe morbidity (Bernatsky et al., 2006). Previous studies have found that several factors, such as genetic, environmental, hormonal, and certain medicines (Pan et al., 2019; Teruel & Alarcón‐Riquelme, 2016; Tsokos, 2011), may be responsible for this disorder, but the pathogenesis of SLE remains incompletely understood.

Recent years, epigenetic regulation that is involved in the modification of DNA and proteins has been reported to contribute significantly to SLE (Wang, Zhang, Wu, Chen, & Shi, 2018; Zhu et al., 2016), but RNA modifications in SLE are still poorly investigated (Hedrich, 2017). N6‐methyladenosine (m6A) is the most prevalent and evolutionarily conserved modification, occurring in nearly all types of RNAs and in most organisms (Wang et al., 2014). In the dynamic regulation of m6A modification, m6A methyltransferases, demethylases, and RNA‐binding proteins play crucial roles. In fact, it has been shown that methyltransferase‐like 3 (METTL3), methyltransferase‐like 14 (METTL14), and Wilms tumor 1‐associating protein (WTAP) act as canonical m6A methyltransferases (Liu et al., 2014; Ping et al., 2014; Schwartz et al., 2014). Two members of the a‐ketoglutarate‐dependent dioxygenase protein family, fat mass and obesity‐associated protein (FTO), and a‐ketoglutarate‐dependent dioxygenase alkB homolog 5 (ALKBH5) have been shown as powerful m6A demethylases (Jia et al., 2011; Zheng et al., 2013). Moreover, YT521‐B homology domains 2 (YTHDF2) act as a RNA‐binding protein, can recognize m6A modification, decode the methylation code, and finally transform them into diverse functional signals (Maity & Das, 2016). Accumulating evidence has indicated that dysregulation of m6A modification, m6A methyltransferases, demethylases, and RNA‐binding proteins may be involved in diabetes, infertility, cancers (Shen et al., 2015; Sibbritt, Patel, & Preiss, 2013; Sun, Wu, & Ming, 2019; Yang et al., 2016), supporting the hypothesis that m6A and the methylation modifier including m6A methyltransferases, demethylases, and RNA‐binding proteins have potential to be new prognostic biomarkers, and novel therapy targets of diseases (Zhao et al., 2018). However, little is known about the characteristic of m6A in peripheral blood mononuclear cells (PBMCs) in human SLE. The present study aimed to determine the mRNA expression level of *METTL3*, *MTEEL14*, *WTAP*, *FTO*, *ALKBH5*, and *YTHDF2* in PBMCs from SLE patients using quantitative real‐time polymerase chain reaction (qRT‐PCR), which investigating whether m6A play a role in the occurrence and development of SLE.

## 2 MATERIALS AND METHODS

### 2.1 Patient variables and controls

A total of 54 patients fulfilled the revised American College of Rheumatology criteria for SLE (Tan et al., 1982) were recruited from the First Affiliated Hospital of Nanchang University from 2018.10 to 2019.3. Among them, 49 patients were new‐onset SLE that is first time diagnosis of SLE and no history of immunosuppressive drugs or corticosteroids use before recruitment. Among all new‐onset SLE patients, 8 patients were reexamined after 15 days of regular treatment using glucocorticoids and immunosuppressive agents. In the same time period, 42 healthy controls (HC) who had no inflammatory or autoimmune diseases and genetically unrelated to the patients with SLE were enrolled from the First Affiliated Hospital of Nanchang University. The demographic characteristics of the study population are demonstrated in Table 1. The study was approved by the Ethics Committee of the First Affiliated Hospital of Nanchang University (approval no. 2014003) and was performed according to the Declaration of Helsinki. All participants provided signed informed consent before they entered the study.

**Table 1:** Clinical characteristics of SLE patients and HC

Categories	SLE patients (*n* = 54)	HC (*n* = 42)
Females, %	90.74	83.33
Age, mean (*SD*), years	36.00 ± 16.68	40.60 ± 13.17
SLEDAI score, mean (*SD*)	13.45 ± 8.70	
Anti‐dsDNA(IU/mL), mean (*SD*)	336.88 ± 449.79	
Anti‐ENA (49 patients)		
Anti‐Sm, %	30.61	
Anti‐nRNP/Sm, %	53.06	
Anti‐RIB‐P, %	36.73	
Anti‐nucleosome, %	40.82	
Anti‐SSA, %	69.39	
Anti‐SSB, %	16.33	
C3 (g/L), mean (*SD*)	0.59 ± 0.312	
C4 (g/L), mean (*SD*)	0.15 ± 0.12	
IgG (g/L), mean (*SD*)	18.78 ± 5.38	
ESR (mm/h), mean (*SD*)	60.19 ± 35.79	
CRP (mg/L), mean (*SD*)	17.75 ± 31.92	
WBC (10^9^/L), mean (*SD*)	6.11 ± 3.61	5.78 ± 0.90
RBC (10^12^/L), mean (*SD*)	3.72 ± 0.80*	4.59 ± 0.39
HGB (g/L), mean (*SD*)	116.65 ± 75.49*	136.05 ± 21.14
HCT (L/L), mean (*SD*)	0.33 ± 0.07*	0.41 ± 0.03
PLT (10^9^/L), mean (*SD*)	200.14 ± 106.27*	246.24 ± 53.94
Lymphocytes (10^9^/L), mean (*SD*)	1.25 ± 0.62*	1.86 ± 0.30
Lymphocytes (%), mean (*SD*)	23.38 ± 10.43*	32.51 ± 5.20
Monocytes (10^9^/L), mean (*SD*)	0.48 ± 0.39	0.35 ± 0.09
Monocytes (%), mean (*SD*)	7.71 ± 3.31*	6.14 ± 1.41
Neutrophils (10^9^/L), mean (*SD*)	4.32 ± 3.12	3.45 ± 0.74
Neutrophils (%), mean (*SD*)	67.81 ± 11.70*	59.13 ± 5.52
NLR, mean (*SD*)	4.29 ± 3.86*	1.89 ± 0.48
PLR, mean (*SD*)	192.34 ± 134.73	134.95 ± 33.76
Clinical features		
Fever, %	42.00	
Rash, %	38.00	
Alopecia, %	28.00	
Arthritis, %	54.00	
NPLE, (%)	6.00	
Ulceration, %	8.00	
Pleuritis, %	37.50	
Pericarditis, %	34.69	
LN, %	50.00	

Abbreviations: Anti‐dsDNA, Anti‐double‐stranded DNA; Anti‐ENA, Anti‐extractable nuclear antigen; Anti‐nRNP/Sm, Anti‐nuclear ribonucleoprotein/Smith antibody; Anti‐RIB‐P, Anti‐ribosomal P‐protein antibody; Anti‐Sm, Anti‐Smith antibody; Anti‐SSA, Anti‐sjögren syndrome A antigen antibody; Anti‐SS‐B, Anti‐sjögren syndrome B antigen antibody; C3, Complement 3; C4, Complement 4; CRP, C‐reactive protein; ESR, Erythrocyte sedimentation rate; HC, Controls; HCT, Hematocrit; HGB, Hemoglobin; IgG, Immunoglobulin G; L%, Lymphocyte percentage; L, Lymphocyte count; LN, Lupus nephritis; M%, Monocyte percentage; M, Monocyte count; N%, Neutrophil percentage; N, Neutrophil count; NLR, Neutrophil–lymphocyte ratio; NPLE, Neuropathic lupus erythematosus; PLR, Platelet–lymphocyte ratio; PLT, Platelet count; RBC, Red blood cell count; SLE, Systemic lupus erythematosus; SLEDAI, SLE disease activity index; WBC, White blood cell count.

* *p* < .05 SLE compared to HC.

### 2.2 PBMCs samples collection and total RNA extraction

The samples collection and total RNA extraction are consistent with our previous research (Luo et al., 2019).

### 2.3 Quantitative real‐time polymerase chain reaction analysis (QRT‐PCR) analysis

Complementary DNA (cDNA) was acquired from blood samples by reverse transcription using a PrimeScript™ RT reagent kit (Takara Bio Inc, Japan). *METTL3* (NM_019852.5), *METTL14* *(NM_201638.2)*, *WTAP* (NM_001270531.2), *ALKBH5* (NM_017758.4), *FTO* (NM_001080432.3), and *YTHDF2* (NM_001172828.2) transcripts were quantified by an ABI 7500 Real‐time PCR System (Applied Biosystems; Thermo Fisher Scientific, Inc.) using SYBR^®^ Premix Ex Taq™ II (Takara Bio Inc). The amplification primers sequences for *METTL3*, *METTL14*, *WTAP*, *ALKBH5*, *FTO*, *YTHDF2*, and GAPDH are listed in Table 2. The relative expression of circRNAs was derived by 2^−∆Ct^ method (Zhang et al., 2016).

**Table 2:** The amplification primers sequences

Gene name	Sequence (5′−3′)
*METTL3*	F: AAGCTGCACTTCAGACGAAT
	R: GGAATCACCTCCGACACTC
*METTL14*	F: AGAAACTTGCAGGGCTTCCT
	R: TCTTCTTCATATGGCAAATTTTCTT
*WTAP*	F: GGCGAAGTGTCGAATGCT
	R: CCAACTGCTGGCGTGTCT
*ALKBH5*	F: CCCGAGGGCTTCGTCAACA
	R: CGACACCCGAATAGGCTTGA
*FTO*	F: TGGGTTCATCCTACAACGG
	R: CCTCTTCAGGGCCTTCAC
*YTHDF2*	F: GGCAGCACTGAAGTTGGG
	R: CTATTGGAAGCCACGATGTTA
GAPDH	F: TGCACCACCAACTGCTTAGC
	R: GGCATGGACTGTGGTCATGAG

Note

Methyltransferase‐like 3 (*METTL3*), Methyltransferase‐like 14 (*METTL14*), Wilms tumor 1‐associating protein (*WTAP*), A‐ketoglutarate‐dependent dioxygenase alkB homolog 5 (*ALKBH5*), Fat mass and obesity‐associated protein (*FTO*), YT521‐B homology domains 2 (*YTHDF2*).

*METTL3* (NM_019852.5), *METTL14* (NM_201638.2), *WTAP* (NM_001270531.2), *ALKBH5* (NM_017758.4), *FTO* (NM_001080432.3), *YTHDF2* (NM_001172828.2).

### 2.4 Statistical analysis

Student's *t* test or Mann–Whitney *U*‐test were used to compare the data according to the normality. The Spearman method was used for correlation analysis. Logistic regression analysis was used for evaluating risk factor. All the data were analyzed by GraphPad Prism version 5.0 (GraphPad Software) and SPSS version 17.0 (SPSS Inc.). *p* values ≤.05 were considered statistically significant.

## 3 RESULTS

### 3.1 Screening of abnormal expression *METTL3*, *METTL14*, *WTAP*, *ALKBH5*, *FTO*, and *YTHDF2* in PBMCs from SLE patients and HC

**Figure 1:**
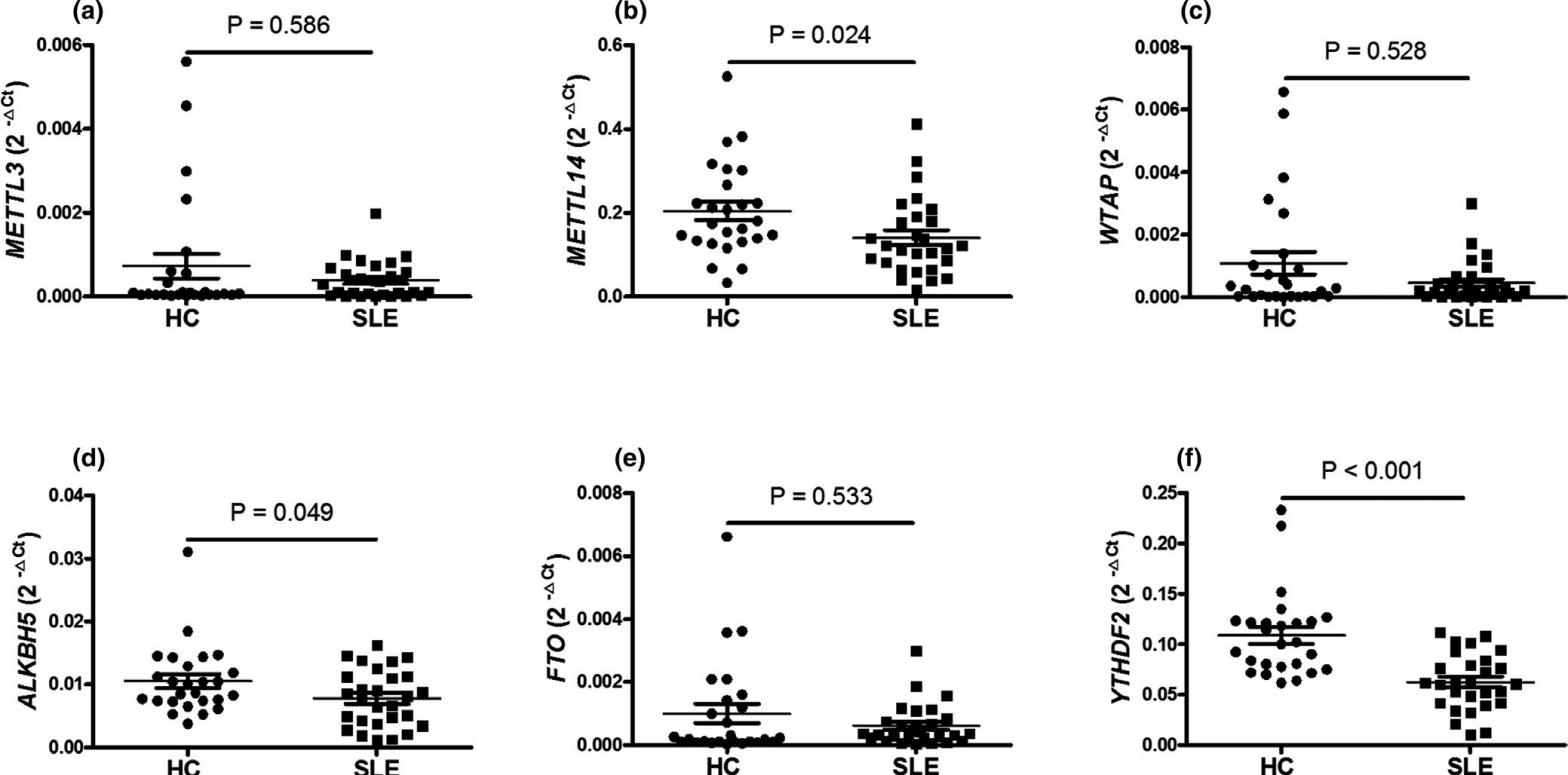
Screening of abnormal expression *METTL3*, *METTL14*, *WTAP*, *ALKBH5*, *FTO*, and *YTHDF2* in PBMCs from SLE patients and HC. The mRNA expression of methyltransferase‐like 3 (*METTL3*), methyltransferase‐like 14 (*METTL14*), wilms tumor 1‐associating protein (*WTAP*), a‐ketoglutarate‐dependent dioxygenase alkB homolog 5 (*ALKBH5*), fat mass and obesity‐associated protein (*FTO*), and YT521‐B homology domains 2 (*YTHDF2*) in peripheral blood mononuclear cells (PBMCs) from 28 systemic lupus erythematosus (SLE) patients and 26 healthy controls (HC) were detected by quantitative reverse transcription‐polymerase chain reaction (qRT‐PCR). The data for *METTL3* (a), *METTL14* (b), *WTAP* (c), *ALKBH5* (d), *FTO* (e), and *YTHDF2* (f) were shown. Each dot represents an individual patient. Difference was evaluated with Student's *t*‐test or Mann–Whitney *U*‐test. *p* < .05 indicates a significant difference. *METTL3* (NM_019852.5), *METTL14* (NM_201638.2), *WTAP* (NM_001270531.2), *ALKBH5* (NM_017758.4), *FTO* (NM_001080432.3), and *YTHDF2* (NM_001172828.2)

To investigative the expression of *METTL3*, *METTL14*, *WTAP*, *ALKBH5*, *FTO*, and *YTHDF2* in patients with SLE and HC, PBMCs from 28 SLE patients and 26 age‐ and sex‐matched HC were collected to detect their expression using qRT‐PCR. As shown in Figure 1, the expression of *METTL14*, *ALKBH5*, and *YTHDF2* in PBMCs from SLE patients were significantly decreased than that in HC (all *p* < .050), while the expression of *METTL3*, *WTAP*, and *FTO* in PBMCs from SLE patients and HC was unchanged (all *p* > .050).

### 3.2 Validation of *METTL14*, *ALKBH5*, and *YTHDF2* expression in PBMCs

**Figure 2:**
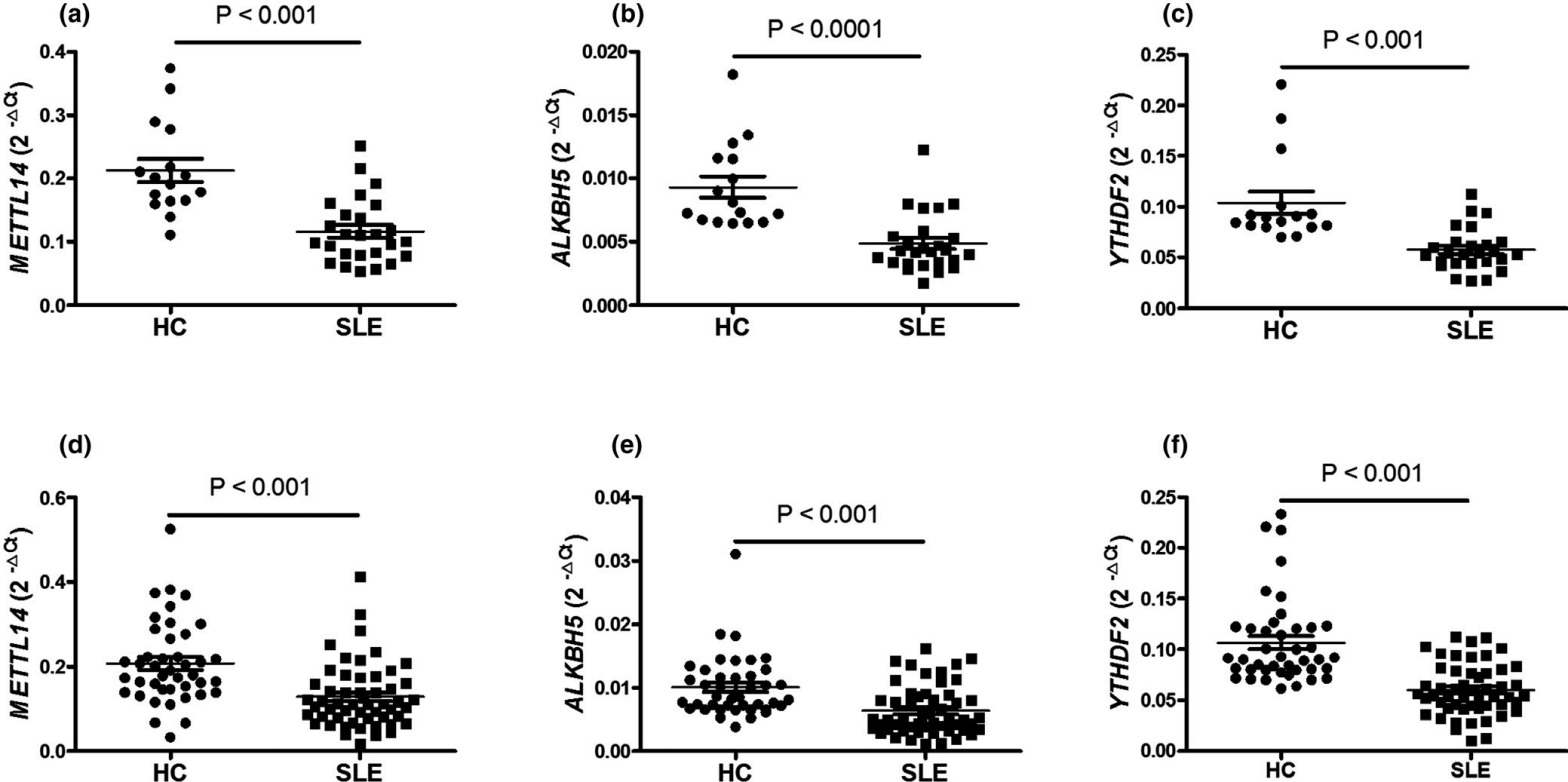
Validation of *METTL14*, *ALKBH5*, and *YTHDF2* expression in PBMCs. The mRNA expression of methyltransferase‐like 14 (*METTL14*), a‐ketoglutarate‐dependent dioxygenase alkB homolog 5 (*ALKBH5*), and YT521‐B homology domains 2 (*YTHDF2*) in peripheral blood mononuclear cells (PBMCs) from an independent validation testing set including 26 systemic lupus erythematosus (SLE) patients, 16 healthy controls (HC), and all the SLE patients, HC were detected by quantitative reverse transcription‐polymerase chain reaction (qRT‐PCR). The data for *METTL14* (a), *ALKBH5* (b), and *YTHDF2* (c) in an independent validation testing set and *METTL14* (d), *ALKBH5* (e), and *YTHDF2* (f) in all subjects were shown. Each dot represents an individual patient. Differences were evaluated with Student's *t* test or Mann–Whitney *U*‐test. *p* < .05 indicates a significant difference. *METTL14* (NM_201638.2), *ALKBH5* (NM_017758.4), and *YTHDF2* (NM_001172828.2)

To verify the results in the screening set, an independent validation testing set consisting of 26 SLE patients and 16 HC were enrolled and their expression was determined. Similar to the screening set, the results demonstrated that the expression of *METTL14*, *ALKBH5*, and *YTHDF2* was all significantly decreased in PBMCs from SLE patients compared with HC (all *p* < .0001) (Figure 2a–c). From the data of all the SLE patients and HC, the expression of *METTL14*, *ALKBH5*, and *YTHDF2* in PBMCs from 54 SLE patients was significantly decreased compared to 42 HC (all *p* < .0001) (Figure 2d–f).

### 3.3 Correlation between *METTL14*, *ALKBH5*, and *YTHDF2* in PBMCs and clinical data in SLE patients

**Figure 3:**
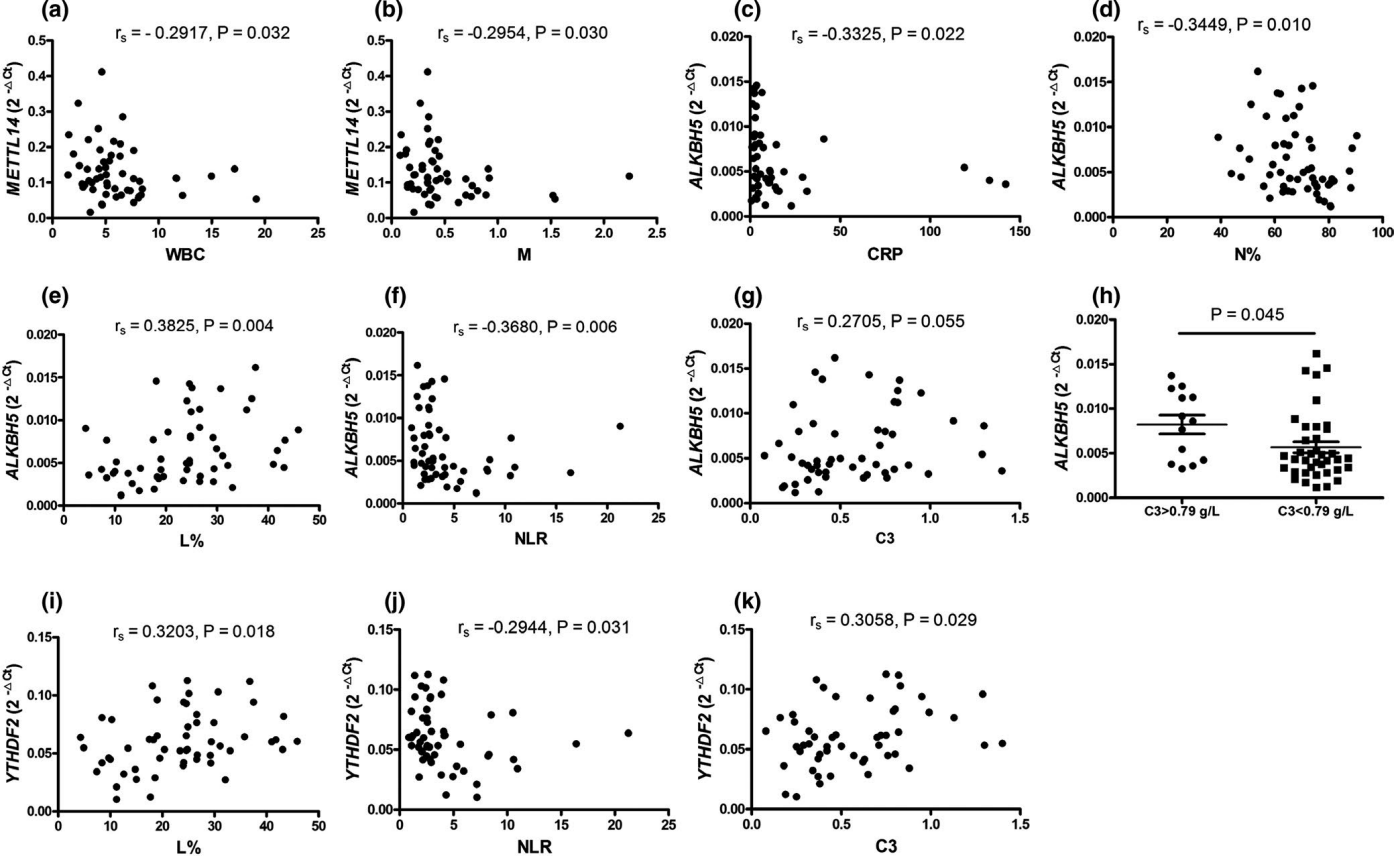
Correlation between *METTL14*, *ALKBH5*, and *YTHDF2* in PBMCs and laboratory data in SLE patients. Complement 3 (C3), C‐reactive protein (CRP), lymphocyte percentage (L%), monocyte count (M), neutrophil percentage (N%), neutrophil–lymphocyte ratio (NLR), and white blood cell count (WBC) were measured. Correlations between the mRNA expression of methyltransferase‐like 14 (*METTL14*) and WBC (a) or *M* (b), the mRNA expression of a‐ketoglutarate‐dependent dioxygenase alkB homolog 5 (*ALKBH5)* and CRP (c) or N% (D) or L% (e) or NLR (F) or C3 (g, h), the mRNA expression of YT521‐B homology domains 2 (*YTHDF2*) and L% (i) or NLR (j) or C3 (k) were analyzed. Each dot represents an individual patient. Correlations were evaluated with Spearman's nonparametric test. Differences were evaluated with Student's *t* test. *p* < .05 indicates a significant difference. *METTL14* (NM_201638.2), *ALKBH5* (NM_017758.4), and *YTHDF2* (NM_001172828.2)

The correlation test was analyzed to evaluate the relationship between the clinical data of SLE (SLEDAI, CRP, ESR, IgG, C3, C4, WBC, RBC, HGB, HCT, PLT, L, M, N, NLR, PLR, antibodies, and drug therapy) and the expression of peripheral blood *METTL14*, *ALKBH5*, and *YTHDF2*. As shown in Figure 3, the expression of *METTL14* in PBMCs from SLE patients correlated with WBC (*r_s_* = −.2917, *p* = .032), *M* (*r_s_* = −.2954, *p* = .030), the expression of *ALKBH5* in PBMCs from SLE patients was associated with CRP (*r_s_* = −.3325, *p* = .022), N% (*r_s_* = −.3449, *p* = .010), L% (*r_s_* = .3825, *p* = .004), NLR (*r_s_* = −.3680, *p* = .006), C3 (*p* = .045), and the expression of *YTHDF2* in PBMCs from SLE patients correlated with L% (*r_s_* = .3203, *p* = .018), NLR (*r_s_* = −.2944, *p* = .031), C3 (*r_s_* = 3,058, *p* = .029). However, there was no correlation between the expression of *METTL14*, *ALKBH5*, and *YTHDF2* with other clinical data of SLE.

**Figure 4:**
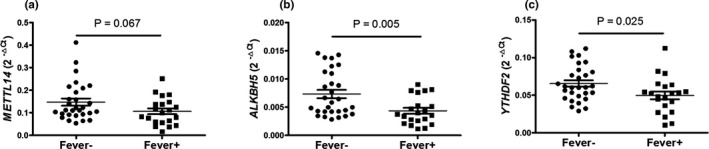
Association between *METTL14*, *ALKBH5*, and *YTHDF2* in PBMCs and fever in SLE patients. Association between the mRNA expression of methyltransferase‐like 14 (*METTL14*) (a), a‐ketoglutarate‐dependent dioxygenase alkB homolog 5 (*ALKBH5)* (b), YT521‐B homology domains 2 (*YTHDF2*) (c), and fever were analyzed. Each dot represents an individual patient. Differences were evaluated with Student's *t* test or Mann–Whitney *U*‐test. *p* < .05 indicates a significant difference. *METTL14* (NM_201638.2), *ALKBH5* (NM_017758.4), and *YTHDF2* (NM_001172828.2)

Subsequently, we evaluated the relationship between the clinical symptoms of SLE including LN, NPLE, arthritis, fever, rash, alopecia, ulceration, pleuritis, pericarditis, and the expression of *METTL14*, *ALKBH5*, and *YTHDF2* in PBMCs. As shown in Figure 4, the expression of *ALKBH5* and *YTHDF2* in PBMCs from SLE patients with fever was significantly decreased than that in SLE patients without fever (all *p* < .050), while the expression of *METTL14* in PBMCs from SLE patients with fever trends to reduce, but a significant difference was not reached (*p* = .067). In addition, there was no association between the expression of *METTL14*, *ALKBH5*, and *YTHDF2* with other clinical symptoms of SLE.

### 3.4 Correlation between these genes in PBMCs from SLE patients

**Figure 5:**
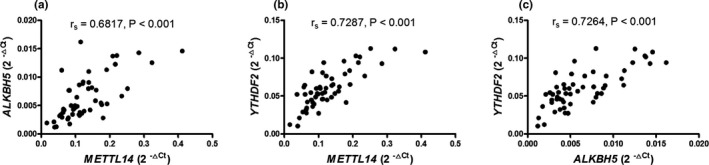
Correlation between these genes in PBMCs from SLE patients. Correlation between methyltransferase‐like 14 (*METTL14*) and a‐ketoglutarate‐dependent dioxygenase alkB homolog 5 (*ALKBH5*) (a) or YT521‐B homology domains 2 (*YTHDF2*) (b), and *ALKBH5* and *YTHDF2* (c) were analyzed. Each dot represents an individual patient. Correlations were evaluated with Spearman's nonparametric test. *p* < .05 indicates a significant difference. *METTL14* (NM_201638.2), *ALKBH5* (NM_017758.4), and *YTHDF2* (NM_001172828.2)

As shown in Figure 5, the expression of *METTL14* in PBMCs from SLE patients positively correlated with *ALKBH5* (*r_s_* = .6817, *p* < .001), *YTHDF2* (*r_s_* = .7287, *p* < .001), and the expression of *ALKBH5* in PBMCs from SLE patients positively correlated with *YTHDF2* (*r_s_* = .7268, *p* < .001).

### 3.5 Decreased mRNA expression of *YTHDF2* in PBMCs was a risk factor for SLE

The aforementioned results demonstrate that the decreased expression of *METTL14*, *ALKBH5*, and *YTHDF2* in PBMCs were associated with SLE pathogenesis. Thus, to investigate whether the expression of *METTL14*, *ALKBH5*, and *YTHDF2* in PBMCs were risk factors for SLE, the “enter method” of logistic regression was used. As shown in Table 3, we acquired the equation about the expression of *METTL14*, *ALKBH5*, and *YTHDF2* in PBMCs, *Y* = −1.112 X1(*METTL14)* *+* 201.063 X2(*ALKBH5)* − 81.543 X3(*YTHDF2)* *+* 5.242, and only the decreased expression of *YTHDF2* in PBMCs was a risk factor for SLE (*p* < .001).

**Table 3:** The expression of *METTL14*, *ALKBH5*, and *YTHDF2* in equation

	*B*	*SE*	Wald	*df*	*p*	Exp (*B*)
*METTL14*	−1.112	4.639	0.057	1	.811	0.329
*ALKBH5*	201.063	116.248	2.992	1	.084	2.092E87
*YTHDF2*	−81.543	21.202	14.791	1	<.001	0.000
Constant	5.242	1.114	22.139	1	<.001	189.135

Note

Methyltransferase‐like 14 (*METTL14*), A‐ketoglutarate‐dependent dioxygenase alkB homolog 5 (*ALKBH5*), YT521‐B homology domains 2 (*YTHDF2*).

*METTL14* (NM_201638.2), *ALKBH5* (NM_017758.4), *YTHDF2* (NM_001172828.2).

## 4 DISCUSSION

It is well known that epigenetic alterations, such as DNA methylation and histone modifications, have been demonstrated to play an important role in SLE progression (Renaudineau & Youinou, 2011). Zhu et al. (2016) combined DNA methylation analysis and whole‐genome transcription of PBMCs to identify epigenetic biomarkers for SLE, and demonstrated the effects of DNA methylation on differential genes involved in IFN, TLR signaling pathways, and inflammatory cytokines, suggesting aberrant DNA methylation may be relevant to the pathogenesis of SLE. Recently, N6‐methyladenosine (m6A) is the most common form of mRNA modification, and is dynamically regulated by the m6A RNA methylation regulators. A study conducted by Zheng, Hou, Zhou, Li, & Cao (2017) highlights the key role of m6A modification (*ALKBH5*) in DDX46‐mediated inhibition of production of type I interferon after viral infection. Li et al. (2017) and Lichinchi et al. (2016) indicated *ALKBH5* and *METTL3*, *METTL14* implicated in T‐cell response and T‐cell differentiation, homeostasis, respectively. Although considering the fact that type I interferon and T‐cell‐mediated immune response are associated with SLE pathogenesis, Li, Fan, Leng, Pan, and Ye (2018) speculate that m6A modification may justify the pathogenesis of SLE and take part in the initiation and progression of SLE. However, the role of m6A manipulation by RNA methylation regulators in SLE has not been studied. In this study, the mRNA expression levels of *METTL3*, *MTEEL14*, *WTAP*, *FTO*, *ALKBH5*, and *YTHDF2* in PBMCs from SLE patients and HC were detected, and we found that the mRNA expression levels of *MTEEL14*, *ALKBH5*, and *YTHDF2* in PBMCs from SLE patients were significantly decreased than HC, suggesting that the down expression of *MTEEL14*, *ALKBH5*, and *YTHDF2* in PBMCs in SLE may affect the m6A modification of some genes in SLE, and then promote the development of SLE.

Patients with SLE have a complex array of abnormalities involving their immune system. Hematologic disorders are one of the most frequent types of disorder in patients with SLE (Vanarsa et al., 2012; Verma, Arora, Marwaha, Kumar, & Das, 2005) and are associated with disease activity in SLE. Ma, Zeng, Chen, Chen, and Zhou (2019) indicated that NLR was significantly higher in SLE patients compared with HC, and was positively correlated with SLEDAI. And C3 is used as a classical activity biomarkers for SLE. In this study, we investigated the correlations between the expression of *METTL14*, *ALKBH5*, and *YTHDF2* and clinical data in SLE patients, and results showed that the down expression of *METTL14* was associated with WBC, M, CRP, and fever, the down expression of *ALKBH5* was associated with N%, L%, NLR, C3, and fever, and the down expression of *YTHDF2* was inversely associated with L%, NLR, C3, and fever, suggesting downexpression of *METTL14*, *ALKBH5*, and *YTHDF2* was associated with disease activity in SLE.

There is increasing evidence that serum biomarkers can provide reliable information about the course of clinical conditions characterized by immune and inflammatory responses (Di Napoli et al., 2018; Lattanzi et al., 2019; Lattanzi, Di Napoli, Ricci, & Divani, 2020). And, our results showed a direct relationship between these serum biomarkers and alterations in mRNA expression (*METTL14*, *ALKBH5*, and *YTHDF2*), suggesting easily obtainable and highly informative laboratory parameters can be used to monitor the course of SLE patients and *METTL14*, *ALKBH5*, and *YTHDF2* may be used as routine clinical parameters.

In a recent study, demethylases FTO have been demonstrated to positively correlate with methyltransferases methyltransferase complex (*METTL3*, *METTL14*, and *WTAP*) in patients with type 2 diabetes (Yang et al., 2019). In other study, the expression level of RNA‐binding protein (*YTHDF*) displayed a positive correlation with methyltransferases methyltransferase complex (*METTL3*, *METTL14*, and *WTAP*) (Zhang et al., 2019). And our results showed that the expression of *METTL14* in PBMCs from SLE patients positively correlated with *ALKBH5*, *YTHDF2*, and the expression of *ALKBH5* in PBMCs from SLE patients positively correlated with *YTHDF2*. Thus, previous studies and this study manifested the collaboration among writers (*METTL14*)–erasers (*ALKBH5*)–readers (*YTHDF2*) sets up the m6A threshold and perturbs that m6A threshold, leading to uncontrolled expression/activity of virulence gene, resulting in the occurrence and development of disease (Panneerdos et al., 2018).

It is worth noting that YTHDF2 knockdown significantly increased the LPS‐induced IL‐6, TNF‐α, IL‐1β, and IL‐12 expression and the phosphorylation of p65, p38, and ERK1/2 in NF‐κB and MAPK signaling (Yu, Li, Feng, Cai, & Xu, 2019), which play important roles in SLE pathogenesis (Brightbill et al., 2018; Garcia‐Rodriguez et al., 2012). In this study, we found that decreased mRNA expression of *YTHDF2* in PBMCs was a risk factor for SLE, suggesting decreased mRNA expression of *YTHDF2* may activate MAPK and NF‐κB signaling pathways, which promote the occurrence and development of SLE.

## 5 CONCLUSIONS

Data in the current study show that SLE patients had lower mRNA expression of *METTL14*, *ALKBH5*, and *YTHDF2* than HC. This decreased mRNA expression of *METTL14* was associated with WBC and M; this decreased mRNA expression of *ALKBH5* was associated with CRP, N%, L%, NLR, C3, and fever; and the decreased mRNA expression of *YTHDF2* was associated with L%, NLR, C3, and fever. In addition, there was a positive correlation between mRNA expression of *METTL14*, *ALKBH5*, and *YTHDF2* in PBMCs from SLE patients. Importantly, logistic regression analysis revealed that decreased mRNA expression of *YTHDF2* was a risk factor for SLE. Taken all together, our findings suggest decreased *YTHDF2* that was associated with disease activity may play an important role in the pathogenesis of SLE, *METTL14* and *ALKBH5* may be concomitantly decreased.

## CONFLICT OF INTEREST

The authors declare no conflicts of interest.

## AUTHORS' CONTRIBUTIONS

All authors were involved in revising the text critically for important intellectual content, and all authors approved the final version to be published. QL and JML are responsible for the study conception and design. JYR, LZ, BQF, and YG collected the samples and clinical parameters. QL, JYR, ZKH, and JML are responsible for the analysis and interpretation of data. QL and JML drafted the article.

## Data Availability

The data used to support the findings of this study are available from the corresponding author upon request.
